# Vestibulo-Ocular Reflex Results in Patients with Intralabyrinthine Schwannomas: Case Series with a Literature Review

**DOI:** 10.3390/diagnostics15162093

**Published:** 2025-08-20

**Authors:** Xiaoye Chen, Yingzhao Liu, Yangming Leng, Ping Lei, Xingqian Shen, Kaijun Xia, Qin Liu, Ziying Xu, Bo Liu, Hongjun Xiao

**Affiliations:** 1 Department of Otorhinolaryngology-Head and Neck Surgery, ENT Institute, Union Hospital, Tongji Medical College, Huazhong University of Science and Technology, Wuhan 430022, China; m202376251@hust.edu.cn (X.C.); d202382071@hust.edu.cn (Y.L.); lyangming@foxmail.com (Y.L.); sxq15207158114@163.com (X.S.); xiakaijun626@hust.edu.cn (K.X.); liuqinent@163.com (Q.L.); xzy15871263046@163.com (Z.X.); 2 Hubei Province Clinical Research Center for Deafness and Vertigo, Wuhan 430022, China; 3 Department of Radiology, Union Hospital, Tongji Medical College, Huazhong University of Science and Technology, Wuhan 430022, China; leiping@hust.edu.cn

**Keywords:** intralabyrinthine schwannoma, vestibulo-ocular reflex, vestibular function, video head impulse test, signal intensity

## Abstract

**Background and Clinical Significance:** Intralabyrinthine schwannoma (ILS) is a rare benign tumor of the inner ear, often presenting with nonspecific symptoms such as hearing loss, tinnitus and vertigo. Vestibular function in ILS patients remains underexplored. This study aims to evaluate vestibulo-ocular reflex (VOR) function and inner ear magnetic resonance imaging (MRI) signal changes in ILS, and to provide insights into potential mechanisms underlying vestibular dysfunction. **Case Presentation:** We report four cases of MRI confirmed ILS, including two intravestibular and two intravestibulocochlear schwannomas. All patients exhibited unilateral canal paresis on caloric testing, and two of three showed abnormal video head impulse test (vHIT) with decreased VOR gain and corrective saccades. Decreased signal intensity was observed in the semicircular canals in three cases, in the vestibule in one case, and in the cochlea in one case. A systematic literature review including 10 studies (n = 171) showed a 73.3% rate of abnormal caloric responses. Five studies conducted vHIT, reporting reduced mean VOR gain and corrective saccades, though quantitative analysis was limited. Cervical and ocular vestibular evoked myogenic potential abnormalities were found in 68.4% and 65.7% of reported cases, respectively. **Conclusions**: Impaired VOR function in patients with ILS may result not only from anatomical disruption but also from underlying biochemical or metabolic alterations within the inner ear.

## 1. Introduction

Intralabyrinthine schwannoma (ILS) is a rare, slow-growing benign tumor originating from the distal branches of the vestibulocochlear nerve. Its low prevalence (1.1 per 100,000) and typically small size make diagnosis challenging for both radiologists and otolaryngologists [1]. Clinical manifestations are often nonspecific and may include unilateral hearing loss (95%), episodic vertigo (22–71%), tinnitus (51%), and imbalance (35%), frequently mimicking Ménière’s disease (MD) and other inner ear disorders [2]. The gold standard diagnostic tool for ILS is magnetic resonance imaging (MRI) of the inner ear. These tumors typically appear as filling defects on T2-weighted images and enhance on contrast-enhanced T1-weighted images. Based on tumor location and involved anatomical structures, ILS was initially classified into seven subtypes by Kennedy et al. [3], with intracochlear and intravestibular types being the most common. Building on this, Salzman et al. [4] removed one subtype, and Van Abel et al. [5] added three subtypes to modify the classification system. Recent studies have suggested that the classification and tumor location significantly affect the inner ear function [6].

In addition to classic radiological features of ILS, recent insights from studies on vestibular schwannoma (VS) have shown that decreased signal intensity in the inner ear on heavily T2-weighted MRI may reflect potential pathological changes in labyrinthine fluid. These include elevated protein concentration, impaired endolymphatic circulation, or disruption of the blood–labyrinth barrier [7,8]. While such findings have been well-documented in VS, their occurrence in ILS has not been studied. Given the anatomical continuity and overlapping pathophysiological characteristics between VS and ILS, it is reasonable to hypothesize that similar signal intensity changes may occur in ILS as well. Identifying such alterations could provide a reference for understanding inner ear microenvironmental disruptions and informing clinical management strategies.

The vestibulo-ocular reflex (VOR) is a critical physiological mechanism that stabilizes gaze during head movement. Two primary clinical tools used to evaluate VOR are the video head impulse test (vHIT) and caloric testing. High-frequency and physiological head movements are assessed by vHIT, whereas low-frequency responses in the horizontal semicircular canals are evaluated by caloric testing. Combining the results of vHIT and caloric testing provides a comprehensive assessment of VOR function in ILS patients [9]. However, few studies have examined vestibular function in ILS patients. The primary aim of this study was to report our clinical experience with vestibular function assessment and signal intensity of the inner ear in a series of patients diagnosed with ILS. In addition, a literature review was also performed to further explore the VOR abnormalities in ILS and explore potential clinical and neuroradiological mechanisms underlying vestibular dysfunction.

## 2. Materials and Methods

### 2.1. Case Series

This case series includes four patients diagnosed with ILS between 2016 and March 2025. Clinical symptoms and audiological, vestibular, and radiological findings were retrospectively evaluated. Special emphasis was placed on assessing VOR function using the vHIT and caloric testing. The study was approved by the Ethical Committee of Union Hospital, Tongji Medical College, Huazhong University of Science and Technology (Institutional Review Board approval No. 0153, 2025.2.22). Written informed consent was obtained from the patients to publish this paper.

Audiometry was conducted in a soundproof cabin across frequencies ranging from 0.25 to 8 kHz. The pure tone average (PTA) was computed as the mean threshold at 0.5, 1.0, 2.0, 4.0 kHz.

Two-dimensional vHIT was conducted using the ICS Impulse system (GN Otometrics, Taastrup, Denmark) by an experienced technician. Passive, unpredictable head impulses were delivered with low amplitude (5–15°) and high peak velocity (150–250°/s), with a minimum of 20 impulses per direction. Pathological vHIT was defined by the presence of pathological saccades defined by Abrahamsen et al. [10] and a horizontal vHIT gain < 0.8 or a vertical vHIT gain < 0.7.

Caloric testing was performed using infrared videonystagmography (Visual Eyes VNG, Micromedical Technologies, Chatham, IL, USA). Alternating thermal stimulation was applied to each external auditory canal using airflow at 24 °C and 50 °C. An interaural asymmetry of 25% or greater was considered as unilateral vestibular hypofunction.

Cervical vestibular evoked myogenic potential (cVEMP) and ocular vestibular evoked myogenic potential (oVEMP) were recorded using the Eclipse system (Interacoustics A/S, Middelfart, Denmark), with stimulation delivered via air-conducted sound. The standardized procedure ensured a high degree of repeatability in the recordings, consistent with the findings reported in our previously published study [11]. Stimuli consisted of 500 Hz tone bursts at 100 dB HL, presented monaurally through insert earphones with a rise–plateau–fall time of 2-–1–2 ms. The stimulation rate was 5.1 Hz. At least 100 stimuli were averaged during each trial. Abnormal VEMP results were defined as cVEMP asymmetry ratio (AR) ≥ 36%, oVEMP AR ≥ 40%, or absent/unreliable response on the affected side.

All imaging was performed within four days of the initial clinical visit. MRI scans were acquired using a Verio or Magnetom Trio 3T scanner (Siemens, Erlangen, Germany) with a 12-element phased array coil. A 3D sampling perfection with application-optimized contrasts using different flip angle evolutions (3D-SPACE) sequence was used to (1) evaluate the extent of ILS and its anatomical relationship with adjacent inner ear structures; (2) assess bilateral signal intensity in the cochlea, vestibule, and semicircular canals (SCCs); and (3) exclude other inner ear malformations. The 3D SPACE sequence parameters were as follows: repetition time of 1000 msec, echo time of 135 msec, slice thickness of 0.5 mm, field of view of 200 × 200 mm^2^, matrix size of 384 × 384, number of averages of 2, and bandwidth of 289 Hz/Px.

This case series was prepared in accordance with the CARE guidelines and checklist to ensure standardized and transparent reporting of clinical information.

### 2.2. Literature Review

A comprehensive literature review was conducted using the Pubmed, Embase, and Web of Science databases, covering the past 20 years (from 1 January 2005 to 1 March 2025). The primary objective was to identify all studies reporting preoperative vestibular function in patients with ILS. The search terms included: “Intralabyrinthine schwannoma” OR “Intracochlear schwannoma” OR “Intravestibular schwannoma” OR “Intravestibulocochlear schwannoma” AND “vestibular function”. The inclusion criteria were as follows: (i) the article was an original study, not an editorial, letter, or review, and (ii) the study reported imaging-confirmed cases of ILS with vestibular function test using caloric testing or vHIT. The search yielded 26 studies for abstract review, which were assessed subsequently in full-text review. The references of the selected articles were additionally reviewed to identify related studies. Two independent reviewers (C.X. and L.Y.) assessed the original articles and identified 10 articles that met the inclusion criteria. From each study, data were extracted on subtype, clinical presentation, nystagmus, vestibular test results, including caloric testing, vHIT, cVEMP, and oVEMP.

## 3. Results

### 3.1. Case Series Presentation

The main demographic characteristics and baseline vestibular function of the four cases are presented in Table 1.

#### 3.1.1. Case 1

**Figure 1:**
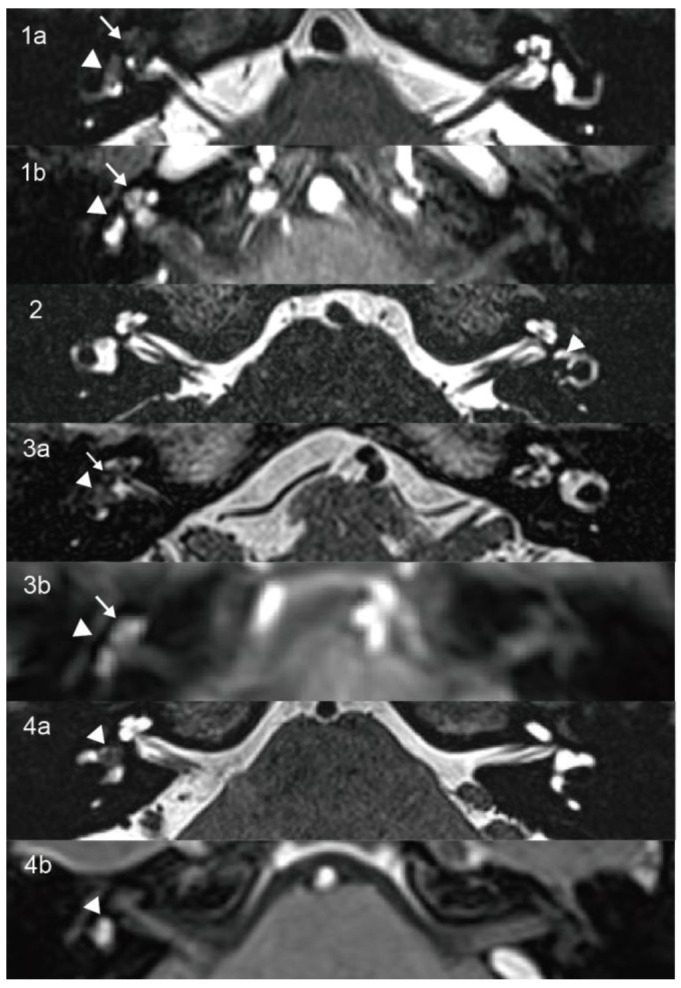
On axial high-resolution T2-weighted MRI (**1a**,**2**,**3a**,**4a**), the lesions appear as hypointense masses that replace the normal fluid signal within the vestibule (arrowhead) and cochlea (arrow) in Cases 1 and 3 and are confined to the vestibule in Cases 2 and 4. Corresponding contrast-enhanced T1-weighted images (**1b**,**3b**,**4b**) show enhancing masses involving both the vestibule and cochlea in Cases 1 and 3 and the vestibule alone in Case 4.

**Figure 2:**
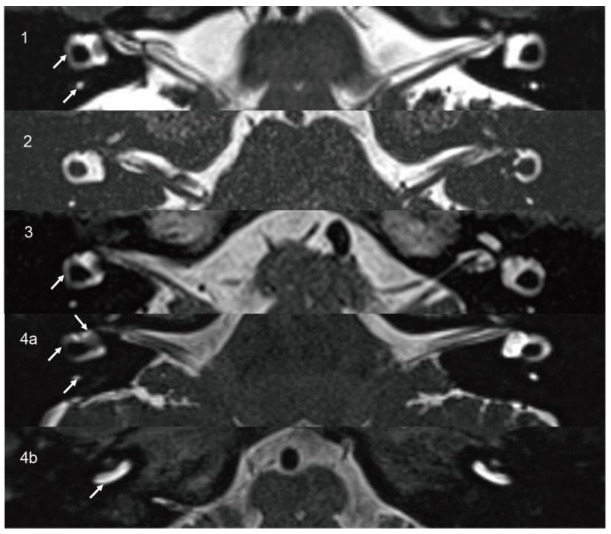
On axial high-resolution T2-weighted MRI (**1**,**3**), decreased signal intensity is observed in SCC on the affected side (arrow) compared to the contralateral side. In Case 2 (**2**), the signal intensity of the cochleae, vestibules, and SCCs appears symmetrical. In Case 4 (**4a,4b**), axial high-resolution T2-weighted MRI demonstrates decreased signal intensity in the vestibule, SCC, and basal turn of the cochlea on the affected side (arrow) compared to the contralateral side. SCC, semicircular canal.

A 60-year-old female presented with years of episodic vertigo, with each episode lasting approximately 30 min. Symptoms had worsened over the previous two months. She also reported bilateral tinnitus and a 10-year history of right-sided sensorineural hearing loss. Her medical history included hypertension and rheumatoid arthritis. No spontaneous nystagmus was observed on physical examination. Following evaluation in our department, the patient underwent audiological, vestibular, and radiological testing. Audiometry revealed complete sensorineural hearing loss in the right ear and mild loss in the left ear. Caloric testing demonstrated a right-sided canal paresis (CP) of 84%. On vHIT, the mean VOR gains were reduced in all three right SCCs, and corrective saccades (CSs) were observed in both the right posterior and horizontal SCCs. MRI showed a hypointense mass in the right vestibule and cochlea on T2-weighted MRI (Figure 1(1a)), with corresponding enhancement on contrast-enhanced T1-weighted imaging (Figure 1(1b)). These findings were consistent with an intravestibulocochlear subtype of ILS. Signal intensity in the right SCCs was decreased relative to the contralateral side (Figure 2(1)). Evaluation of cochlear signal intensity was not possible due to tumor involvement of the cochlea. The patient underwent a follow-up strategy without long-term oral medication. Vertigo resolved spontaneously within one year of onset and has not recurred in the subsequent three years. Hearing in the affected ear slightly deteriorated compared to the initial presentation and affected the ability to follow quiet conversations. Tinnitus persisted but did not interfere with daily activities or quality of life.

#### 3.1.2. Case 2

A 14-year-old male presented with a six-month history of fluctuating left-sided hearing loss and ipsilateral aural fullness. He also reported recurrent episodes of vertigo, each lasting approximately 20 min. Physical examination showed no signs of spontaneous nystagmus. Audiometry revealed moderate sensorineural hearing loss on the affected side, particularly at low frequencies. Caloric testing demonstrated absent response on the left side. The vHIT showed CSs in all three left SCCs. The oVEMP was absent, and the AR of cVEMP was 0.53 with significantly decreased amplitude on the left side. The patient was initially diagnosed with MD and treated with betahistine. After two months of medical therapy, his hearing threshold improved to 15 dB HL. However, T2-weighted MRI revealed a 4.5 × 3 mm hypointense lesion confined to the left vestibule (Figure 1(2)), which was consistent with an intravestibular ILS. Signal intensities in the internal auditory canals, cochleae, vestibules, and SCCs were symmetrical bilaterally (Figure 2(2)). A follow-up approach was adopted for the patient’s management. The patient did not adhere to long-term pharmacological treatment. Hearing in the affected ear showed improvement but did not fully recover. Tinnitus subsided to a mild level and no longer interferes with life quality. Notably, the patient’s vertigo resolved within two years and has not recurred since.

#### 3.1.3. Case 3

A 65-year-old female patient presented with a three-year history of episodic vertigo attacks lasting from several minutes to hours. She also reported right-sided tinnitus and a 30-year history of hearing loss. The physical examination revealed no evidence of spontaneous nystagmus. Audiometry demonstrated complete sensorineural hearing loss in the right ear, with normal hearing on the left. Caloric testing revealed a CP of 42%. The vHIT showed normal VOR gains. The oVEMP was absent on the right, while cVEMP responses were within normal limits. T2-weighted MRI revealed an 8 × 2 mm hypointense mass involving the right vestibule and cochlea (Figure 1(3a)), with corresponding enhancement on contrast-enhanced T1-weighted imaging (Figure 1(3b)). These findings supported a diagnosis of intravestibulocochlear ILS. Signal intensity in the right horizontal SCC was decreased compared to the contralateral side (Figure 2(3)). Due to tumoral involvement, cochlea signal intensity could not be reliably evaluated. We selected conservative management with follow-up. The patient did not receive long-term oral medication and did not experience tinnitus, and no hearing improvement was noted in the right ear. Although vertigo has not recurred, the patient continued to experience occasional episodes of dizziness. Although this causes mild inconvenience in daily activities, it does not affect mood. The self-reported Dizziness Handicap Inventory score was approximately 18, indicating a low level of perceived disability.

#### 3.1.4. Case 4

A 45-year-old female patient presented with a two-year history of right-sided tinnitus and hearing loss, along with three to four episodes of vertigo, each lasting approximately 30 min. She had no relevant medical or family history. No spontaneous nystagmus was observed on physical examination. Audiometry revealed normal hearing in the left ear and moderate sensorineural hearing loss in the right ear, predominantly at low frequencies. Caloric testing showed right-sided areflexia. The oVEMP showed bilateral absence, and the cVEMP was absent on the left side. T2-weighted MRI revealed a hypointense mass in the right vestibule (Figure 1(4a)), which exhibited contrast enhancement on T1-weighted imaging (Figure 1(4b)). These findings were consistent with an intravestibular ILS. Signal intensities in the right SCCs and vestibule (Figure 2(4a)) and the basal turn of the cochlea (Figure 2(4b)) were reduced compared to the contralateral side. A follow-up strategy was deemed appropriate. Hearing in the affected ear has remained stable without further deterioration. The patient reported no tinnitus and did not receive any long-term oral medication. The patient’s vertigo subsided within two years of onset and has remained absent for the past five years.

### 3.2. Literature Review

**Figure 3:**
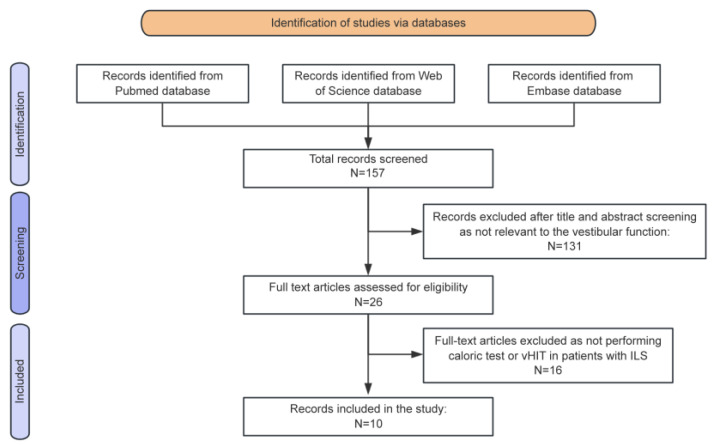
Preferred Reporting Items for Systematic Reviews and Meta-Analyses (PRISMA) diagram summarizing the review process from research to inclusion.

Following the research strategy, 157 manuscripts were initially identified (Figure 3). After the inclusion criteria were applied, 10 articles were included in the final analysis [6,12,13,14,15,16,17,18,19,20]. A summary of the reviewed studies is provided in Table 2. In total, 171 patients were analyzed, comprising 86 males and 85 females, with a mean age of 52.3 years. Among the eight studies that provided detailed audiovestibular symptomatology, data from 150 patients were available. Of these, 141 patients (94.0%) presented with hearing loss, and 101 (67.3%) reported tinnitus. Vestibular symptoms were documented in 84 patients (56.0%), including imbalance, positional vertigo, and MD-like vertigo attacks.

Currently, the gold standard for diagnosing ILS is MRI of the inner ear. Three radiological classification systems were identified and compared (Supplementary Materials). Among the 10 studies reviewed, one adopted Kennedy’s classification, three adopted Salzman’s classification, and one adopted Van Abel’s classification, while the remaining five did not specify the criteria used. Among the seven studies that reported tumor subtypes (n = 169), the common subtypes included intracochlear schwannomas in 79 cases (46.7%), intravestibular in 40 cases (23.7%), intravestibulocochlear in 23 cases (13.6%), and transmodiolar in 19 cases (11.2%).

Vestibular assessment tools included caloric testing, vHIT, cVEMP and oVEMP. Caloric testing was performed in 116 cases, with abnormal findings in 85 cases (73.3%). Among five retrospective studies, three reported detailed vHIT results. In the study by West et al. [12], 20 patients with ILS were evaluated. All cases involving tumors with an intravestibular component (labeled ILSves) showed decreased mean VOR gain. The median VOR gains for the anterior and horizontal SCCs in the ILSves group were 0.50 and 0.40, respectively, compared to 1.52 and 1.02 in patients without vestibular involvement (labeled ILScoch). CSs were observed in 6, 12, and 10 cases of anterior, horizontal, and posterior SCCs, respectively, in the ILS_ves_ subgroup. Lee et al. [13] reported abnormal vHIT findings in 6 of 11 patients for horizontal SCC, 4 of 11 for anterior SCC, and 5 of 11 for posterior SCC. In the study by Plontke et al. [14], five patients underwent vHIT; however, only one case completed horizontal vHIT. The results showed decreased mean VOR gain in 60% of horizontal SCCs and in 75% of anterior and posterior SCCs. Nevertheless, due to the lack of a uniform definition for vHIT abnormality and incomplete reporting of CSs in several studies, it was difficult to determine the precise overall proportion of vHIT abnormalities. There were 38 and 35 cases in which cVEMP and oVEMP were performed, respectively. The abnormal rate was 68.4% for cVEMP and 65.7% for oVEMP.

## 4. Discussion

### 4.1. Impaired VOR Function in ILS

All four patients in our cohort underwent caloric testing, which consistently demonstrated unilateral vestibular hypofunction. The results aligned with previous studies demonstrating a high rate of abnormal caloric responses in ILS. Dubernard et al. [6] reported abnormal caloric function in 77.8% of 110 cases, with 100% abnormality in subtypes involving the vestibule and 57% in cochlear-only subtypes. Similarly, West et al. [12] found abnormal caloric responses in all seven intravestibular and six intravestibulocochlear cases, compared to only 14% in intracochlear lesions. These results support the value of caloric testing, which assesses low-frequency vestibular function in horizontal SCC, in screening VOR integrity in ILS, particularly in subtypes involving the vestibule.

Three patients in our study underwent vHIT to assess high-frequency VOR function. Among them, two demonstrated decreased mean VOR gain and pathological corrective saccades, suggesting significant dysfunction of the involved canals. One patient (No. 3) exhibited normal vHIT results despite having clinical symptoms and caloric hypofunction. To date, few studies have examined vHIT in ILS patients. West et al. [12] evaluated 20 ILS patients and reported reduced mean VOR gain in all cases of ILS_ves_. In this subgroup, 92% exhibited CSs in the horizontal SCCs, and reduced median gains were observed in both the horizontal and anterior SCCs. In contrast, patients with ILS_coch_ mostly demonstrated normal vHIT findings. Similarly, Lee et al. reported an overall vHIT abnormality in 9 out of 14 ILS cases; it affected 36%, 55%, and 45% of the anterior, horizontal, and posterior SCCs, respectively. And patients with vestibular involvement (n = 9) mostly showed abnormal HITs [8/9 (89%)] or canal paresis [8/8 (100%)] [13]. It has been suggested that vHIT is a low-sensitivity, high-specificity test for detecting horizontal VOR pathology compared to the caloric test. This could also explain the vHIT findings in our case No. 3, who demonstrated normal vHIT in the presence of CP of 42%. Collectively, these findings suggest that vHIT could reliably detect severe VOR dysfunction in ILS, which could be complementary to the caloric test.

Interestingly, studies have reported that ILS and MD shared overlapping clinical, audiovestibular, and radiological features, including hearing loss, tinnitus, episodic vertigo, canal paresis, and endolymphatic hydrops (ELH) on Gd-enhanced MRI [2,21]. A feature of MD is the dissociation of caloric test and vHIT results. Studies have proved that most patients with clinically definite MD reported normal vHIT gain but asymmetry in caloric testing [22]. Hannigan et al. even proposed the dissociation as a diagnostic marker for MD [23]. Nonetheless, in contrast to MD, the present findings and prior studies show that more than half of ILS cases exhibit reduced VOR gains on vHIT. The presence of concordant abnormalities in both caloric and vHIT results may therefore strengthen diagnostic confidence for ILS.

### 4.2. Decreased Signal Intensity of Inner Ear in ILS

In our case series, decreased T2-weighted signal intensity was observed in the SCCs in three cases, in the vestibule in one case, and in the basal turn of the cochlea in one case. To our knowledge, no prior studies have reported inner-ear signal alterations in ILS., However, based on findings from VS, it is reasonable to hypothesize that similar mechanisms may underlie these changes in ILS. Previous research on VS has suggested that signal changes on heavily T2-weighted sequences may reflect increased protein content within labyrinthine fluids, potentially secondary to disruption of the blood–labyrinth barrier or tumor-associated exudation [7,8]. In our series, both cases of intravestibulocochlear ILS exhibited reduced signal intensity in the SCCs. Given the tumor’s anatomical proximity to the vestibular end organs, it is plausible that obstruction of fluid pathways, disruption of local homeostasis, or compression of membranous structures may contribute to these imaging findings [24].

### 4.3. Potential Mechanisms of VOR Dysfunction in ILS

In our series, all four patients presented with vertigo and exhibited caloric hypofunction. Each case involved tumors affecting the vestibule, and three demonstrated signal attenuation in the SCCs. The pathogenesis of vestibular hypofunction in ILS patients remains indistinct and multifactorial. Our findings suggest that the anatomical location and subtype of the tumor are important contributors to vestibular dysfunction. However, additional mechanisms are likely involved, as functional impairment has also been observed in intracochlear subtypes [12].

(1) Mechanical compression and anatomical disruption: The subtype and location of the tumor are considered determinants of audiovestibular dysfunction in ILS. It is believed that hearing loss and vestibular hypofunction are the consequences of direct compression or destruction of adjacent inner ear end organs [15,21]. Our cases, all involving vestibular structures, consistently showed abnormal caloric results, supporting this mechanical basis. Nonetheless, vestibular hypofunction is not exclusive to lesions involving the vestibule. West et al. [12] reported significantly reduced mean VOR gain, worse dizziness handicap scores, and decreased caloric response in ILS_ves_ patients, but also noted vestibular hypofunction in ILS_coch_ patients, where tumors were confined to the cochlea. These results may suggest that while mechanical disruption is important, other mechanisms may contribute even in the absence of direct vestibular involvement.
(2) ELH and inner ear fluid imbalance: A second possible mechanism involves the development of ELH. Several studies have described an association between schwannoma and ELH, which may arise due to impaired endolymphatic outflow or altered labyrinthine homeostasis [25,26]. Venkatasamy et al. [25] found that 47% of ILS cases were associated with ipsilateral ELH using non-enhanced high-resolution T2-weighted imaging. In contrast, Pollion et al. [26] observed ELH in only 7% of ILS cases on gadolinium-enhanced MRI. Notably, the majority of Poillon’s cases were intracochlear, whereas subtype data were not specified in Venkatasamy’s study. These discrepancies may reflect differences in imaging modality or tumor subtype. In our series, the two intravestibulocochlear ILS cases showed decreased signal intensity in the SCCs, possibly reflecting altered endolymphatic flow or protein accumulation. These findings are consistent with the hypothesis that tumor compression of the vestibular labyrinth, cochlear duct, or adjacent vasculature may impair endolymph circulation and lead to fluid imbalance. It is also possible that ILS induces subclinical inflammation, contributing to ELH through microvascular changes or epithelial dysfunction [27].
(3) Inflammatory or immune response and microenvironmental alterations: A third potential mechanism involves inflammatory and microenvironmental changes within the labyrinth. Although specific studies on ILS are lacking, findings from VS research provide a plausible framework. In VS, neoplastic Schwann cells have been shown to secrete pro-inflammatory cytokines such as transforming growth factor-β1, interleukin (IL)-1β, IL-6, tumor necrosis factor-α, intracellular adhesion molecule-1, and vascular endothelial growth factor [28,29,30]. These factors can promote tumor proliferation, mediate neural injury, and increase vascular permeability, particularly across the blood–labyrinth barrier, which is a critical regulator of ionic and fluid homeostasis in the inner ear [31]. Although not yet explored in ILS, similar mechanisms may occur at a more localized or subclinical level. Further proteomics and metabolomics of ILS are required to investigate the presence and role of cytokines and barrier integrity in ILS.

This study has several limitations. First, due to the rarity of ILS, the sample size was small, and the findings may not be generalizable to the broader population. Second, as a retrospective study, some test data were incomplete, and oVEMP was performed without bone-conducted vibration, which may have affected sensitivity. Third, all individuals were managed with a wait-and-scan strategy, and none underwent pathological confirmation of the diagnosis. Even so, the combination of detailed clinical history, radiological features, and consistent test findings strongly supports ILS as the most likely diagnosis in each case. In the future, large-scale, multi-center studies incorporating comprehensive audiovestibular assessments and long-term follow-up will be essential to further clarify the pathophysiology of ILS.

## 5. Conclusions

Impaired VOR function in patients with ILS may result not only from anatomical disruption but also from underlying biochemical or metabolic alterations within the inner ear. Concordant abnormalities in caloric and vHIT results, along with signal intensity changes in the adjacent labyrinthine structures, support the hypothesis that ILS may influence a broader microenvironment of the inner ear. Our findings also highlight the importance of combining vestibular testing with advanced imaging to better understand the pathophysiological basis of ILS and guide clinical management.

## Figures and Tables

**Table 1:** Main demographic parameters and baseline vestibular function of the four cases reported.

Case No.	Age (Year)	Sex	Symptoms	Location and Diameter	Subtype	PTA of the Affected Side	Audiogram	vHIT (Anterior/Horizontal/Posterior SCC)	Caloric Test	cVEMP	oVEMP
1	60	F	Recurrent vertigo, tinnitus, HL	Right 3.6 mm	Intravestibulocochlear	>95	PSNHL	Decreased right mean VOR gain and CSs (0.4/0.74/0.35)	CP = 84% right side hypofunction	NP	NP
2	14	M	HL, aural fullness, recurrent vertigo	Left 4.3 mm	Intravestibular	45	LFHL	Decreased left mean VOR gain and CSs (0.7/0.64/0.75)	CP = 100% left side hypofunction	AR = 0.53	Absent
3	65	F	Recurrent vertigo, tinnitus, HL	Right 8 mm	Intravestibulocochlear	>95	PSNHL	Normal (0.74/0.88/0.78)	CP = 42% right side hypofunction	Normal	Absent
4	45	F	Tinnitus, HL, recurrent vertigo	Right 4.3 mm	Intravestibular	48	LFHL	NP	CP = 100% right side hypofunction	Absent on unaffected side	Absent on both sides

AR, asymmetry ratio; CP, canal paresis; F, female; HL, hearing loss; LFHL, low-frequency hearing loss; PTA, pure tone average; M, male; NP, not performed; PSNHL, profound sensorineural hearing loss; SCC: semicircular canal.

**Table 2:** Characteristics of MRI classification and vestibular function of the included original studies.

Authors	N	Radiological Classification Used	Subtypes	Nystagmus	Caloric Test	vHIT
Dubernard et al. [6]	110	Kennedy [3]	21 intravestibular, 55 intracochlear, 13 intravestibulocochlear, 16 transmodiolar, 3 transmacular, 2 tympanolabyrinthine and transotic schwannoma	NP	Abnormal in 56/72	NP
West et al. [12]	20	Salzman [4]	7 intravestibular, 7 intracochlear, 6 intravestibulocochlear schwannoma	NP	Unilateral weakness in ILS_ves_ was 100% and 14% in ILS_coch_	The median gain of horizontal SCC was 0.40 for ILS_ves_ and 1.02 for ILS_coch_. The mean gain of anterior SCC was 0.50 for ILS_ves_ and 1.52 for ILS_coch_. CSs were found in 6, 12, and 10 cases of anterior, horizontal, and posterior SCC, respectively, in ILS_ves_
Lee et al. [13]	16	Van Abel [5]	3 intravestibular, 6 intracochlear, 3 intravestibulocochlear, 2 transmodiolar, 1 transmacular schwannoma	SN was observed in 3/14, vibration-induced nystagmus in 7/13	Unilateral weakness was found in 8/11	Abnormal in 6/11 for horizontal, 4/11 for anterior, and 5/11 for posterior SCC. CSs were not mentioned in detail
Plontke et al. [14]	12	Salzman [4]	3 intravestibular, 6 intracochlear, 1 intravestibulocochlear, 1 transmodiolar, 1 transotic schwannoma	Provocational nystagmus in 1 case	DP range 0–34.7% in 8 patients, 3 cases had DP > 25%	Decreased mean VOR gain in 3/5 for the horizontal, 3/4 for the anterior, and 3/4 for the posterior SCC. CSs were not mentioned in detail
Slattery et al. [15]	8	NM	4 intravestibular, 4 intracochlear schwannoma	Nystagmus in all positions in 1 case	2 cases had caloric symmetry	NP
Machner et al. [16]	1	NM	NM	Without nystagmus	CP range 43–61% in 3 years	Normal
Nishimura et al. [17]	1	Salzman [4] or Kennedy [3]	1 intravestibular schwannoma	Without SN	NP	Decreased VOR gains on anterior and posterior SCC on both sides. CSs were observed on lesion side performed by scleral search coil recordings
Reda et al. [18]	1	NM	1 intracochlear schwannoma	NP	CP = 86%	NP
Schutt et al. [19]	1	NM	NM	NP	Normal	NP
Olsgård [20]	1	NM	1 intravestibular schwannoma	Positional nystagmus	CP = 100%	Decreased VOR gain and CSs on horizontal SCC

AR, asymmetry ratio; CP, canal paresis; CSs, covert saccades; DP, directional preponderance; NM, not mentioned; NP, not performed; SCC, semicircular canal; SN, spontaneous nystagmus; VOR, vestibulo-ocular reflex; ILS_ves_, patients with intravestibular component; ILS_coch_, patients with purely cochlear component.

## Data Availability

The authors confirm that the data supporting the findings of this study are available within the article.

## Supplementary Materials

The following supporting information can be downloaded at https://www.mdpi.com/article/10.3390/diagnostics15162093/s1: Table S1: MRI classification systems for ILS. Figure S1. CARE checklist information for our case series. Key elements for standardized reporting of intralabyrinthine schwannomas cases. Figure S2. The video head impulse test results of three cases performed. A. Case 1 revealed decreased mean VOR gains with corrective saccades on the right side, measuring 0.40, 0.74, and 0.35 for the anterior, horizontal, and posterior semicircular canals, respectively. B. Case 2 demonstrated decreased mean VOR gains with corrective saccades on the left side, with values of 0.70, 0.64, and 0.75 for the anterior, horizontal, and posterior semicircular canals, respectively. C. Case 3 showed normal mean VOR gains of 0.74, 0.88, and 0.78 for the right anterior, horizontal, and posterior semicircular canals, with no corrective saccades observed. *VOR, vestibulo-ocular reflex*.

## Abbreviations

The following abbreviations are used in this manuscript:

3D-SPACE	3D sampling perfection with application-optimized contrasts using different flip angle evolutions
AR	asymmetry ratio
CP	canal paresis
CSs	corrective saccades
ELH	endolymphatic hydrops
ILS	intralabyrinthine schwannoma
MD	Ménière’s disease
MRI	magnetic resonance imaging
VEMP	vestibular evoked myogenic potential
vHIT	video head impulse test
VOR	vestibulo-ocular reflex
SCCs	semicircular canals
