# Supplementary material for: Vestibulo-Ocular Reflex Results in Patients with Intralabyrinthine Schwannomas: Case Series with a Literature Review
Source: Diagnostics (Basel). 2025 Aug 20;15(16):2093. doi: 10.3390/diagnostics15162093 (PMC12385418; doi:10.3390/diagnostics15162093)
Supplement: Supplementary file 1 [file diagnostics-15-02093-s001.zip › diagnostics-3773897-supplementary.pdf]

Supplementary Material

Table S1. MRI classification systems of ILS

Kennedy's classification		Salzman's classification		Van Abel's classification	
Subtypes	Region(s) involved	Subtypes	Region(s) involved	Subtypes	Region(s) involved
Intravestibular	Vestibule ± SCC	Intravestibular	Vestibule ± SCC	Intravestibular	Vestibule ± SCC
Intracochlear	Cochlea	Intracochlear	Cochlea	Intracochlear	Cochlea
Intravestibulocochlear	Vestibule and Cochlea	Vestibulocochlear	Vestibule and Cochlea	Intravestibulocochlear	Vestibule and Cochlea
Transmodiolar	Cochlea + IAC	Transmodiolar	Centered in cochlea with extension through the modiolus into the IAC	Transmodiolar	Cochlea + IAC
Transmacular	Vestibule + IAC	Transmacular	Centered in vestibule with extension into the IAC via the macula cribrosa	Transmacular	Vestibule + IAC
Transotic	Middle ear and vestibule/cochlea + IAC	Transotic	Tumor within the labyrinth with extension into the IAC and middle ear	Transotic	Vestibule ± SCC + cochlea + IAC + middle ear
Tympanolabyrinthine	Middle ear and vestibule/cochlea			Tympanolabyrinthine	Vestibule ± SCC + cochlea + middle ear
				Translabyrinthine	Vestibule ± SCC+ cochlea + IAC
				Involving the CPA	CPA±cochlea±vestibule and/or SCC±IAC±middle ear

		Unspecified	±Cochlea±vestibule ±SCC

CPA: cerebellopontine angle; SCC indicates semicircular canals; IAC, internal auditory canal.

CARE Checklist – 2016: Information for writing a case report

Topic	Item	Checklist item description	Line/Page
**Title**	**1**	The words "case report" should be in the title along with the area of focus	2/1
**Key Words**	**2**	Four to seven key words—include "case report" as one of the key words	35-36/1
**Abstract**	**3a**	Background: What does this case report add to the medical literature?	16-18/1
	**3b**	Case summary: chief complaint, diagnoses, interventions, and outcomes	23-31/1
	**3c**	Conclusion: What is the main "take-away" lesson from this case?	32-34/1
**Introduction**	**4**	The current standard of care and contributions of this case—with references (1-2 paragraphs)	53-74/2
**Timeline**	**5**	Information from this case report organized into a timeline (table or figure)	Not applicable
**Patient Information**	**6a**	De-identified demographic and other patient or client specific information	140,161,179/4;198/5
	**6b**	Chief complaint—what prompted this visit?	140-143,161-163,179-181/;198-200/5
	**6c**	Relevant history including past interventions and outcomes	168-169/4
**Physical Exam**	**7**	Relevant physical examination findings	144,163,181/4;200/5
**Diagnostic Assessment**	**8a**	Evaluations such as surveys, laboratory testing, imaging, etc.	146-154,164-172,182-190/4;201-208/5
	**8b**	Diagnostic reasoning including other diagnoses considered and challenges	150-152,170-171/4;185-188,204-207/5
	**8c**	Consider tables or figures linking assessment, diagnoses and interventions	Figure 1,table 1, supplementary material
	**8d**	Prognostic characteristics where applicable	Not applicable
**Interventions**	**9a**	Types such as life-style recommendations, treatments, medications, surgery	155,173/4;191,209/5
	**9b**	Intervention administration such as dosage, frequency and duration	Not applicable
	**9c**	Note changes in intervention with explanation	Not applicable
	**9d**	Other concurrent interventions	155-156,173-174/4;191-192,209-211/5
**Follow-up and Outcomes**	**10a**	Clinician assessment (and patient or client assessed outcomes when appropriate)	155-159,173-177/4;191-196,209-212/5
	**10b**	Important follow-up diagnostic evaluations	155-159,173-177/4;191-196,209-212/5
	**10c**	Assessment of intervention adherence and tolerability, including adverse events	Not applicable
**Discussion**	**11a**	Strengths and limitations in your approach to this case	369-386/12
	**11b**	Specify how this case report informs practice or Clinical Practice Guidelines (CPG)	280-282/10;306-309,311-322/11
	**11c**	How does this case report suggest a testable hypothesis?	311-368/11
	**11d**	Conclusions and rationale	380-386/12
**Patient Perspective**	**12**	When appropriate include the assessment of the patient or client on this episode of care	Not applicable
**Informed Consent**	**13**	Informed consent from the person who is the subject of this case report is required by most journals	83-84/2;399-401/13
**Additional Information**	**14**	Acknowledgement section; Competing Interests; IRB approval when required	81-83/2;369-398/12

Figure S1. CARE checklist information for our case series. Key elements for standardized reporting of intralabyrinthine schwannomas cases.

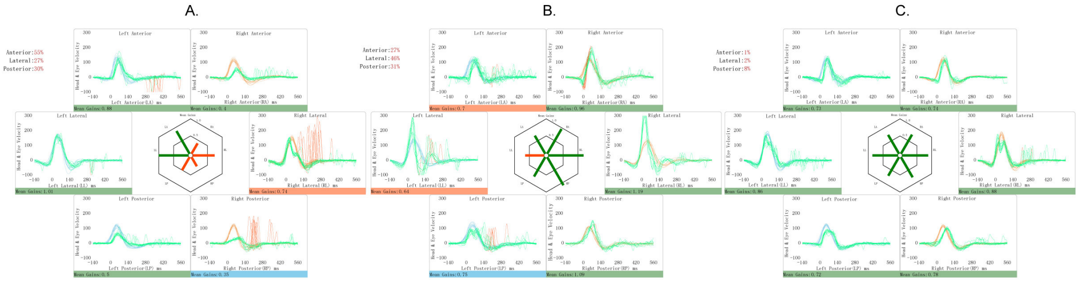

Figure S2. The video head impulse test results of three cases performed. A. Case 1 revealed decreased mean VOR gains with corrective saccades on the right side, measuring 0.40, 0.74, and 0.35 for the anterior, lateral, and posterior semicircular canals, respectively. B. Case 2 demonstrated decreased mean VOR gains with corrective saccades on the left side, with values of 0.70, 0.64, and 0.75 for the anterior, lateral, and posterior semicircular canals, respectively. C. Case 3 showed normal mean VOR gains of 0.74, 0.88, and 0.78 for the right anterior, lateral, and posterior semicircular canals, with no corrective saccades observed. *VOR, vestibulo-ocular reflex*
